# Advertising and large language models: a new frontier influencing medical practice

**DOI:** 10.1038/s41433-026-04518-w

**Published:** 2026-05-09

**Authors:** Mason Crossman, Jeffrey Weng, Stephen Bacchi, Weng Onn Chan

**Affiliations:** 1 Royal Prince Alfred Hospital, Camperdown, NSW Australia; 2 Department of Medicine, University of Sydney, Camperdown, NSW Australia; 3 St Vincents Hospital, Fitzroy, VIC Australia; 4 Faculty of Health and Medical Sciences, Adelaide University, Adelaide, SA Australia; 5 Lyell McEwin Hospital, Elizabeth Vale, SA Australia

**Keywords:** Health services, Scientific community, Business and industry

Large Language Models (LLMs) have infiltrated healthcare and are already shaping how patients choose clinicians, investigations, and treatments. Advertising, whether overt or subtle, may affect patient choice through LLMs. This is of particular concern, as patients are becoming increasingly reliant on and trusting of medical advice provided by these systems [1].

In ophthalmology, LLMs are already used to summarise evidence, draft correspondence, augment triage systems and answer questions from patients and clinicians [2, 3]. Unlike traditional search engines, LLMs do not simply retrieve and rank information [4]. Given search engine optimisation (SEO) influences visibility whilst LLMs influence interpretation, there is a concerning possibility that information ecosystems could be engineered not only for information to be ranked highly, but also to be preferentially synthesised into seemingly authoritative medical advice [4].

Traditional search engines have preserved a buffer by allowing users to compare multiple sources before deciding what to trust. LLMs, however, compress this process into a single narrative response, reducing the user’s ability to independently evaluate sources. Google’s AI overview has already embedded AI-generated summaries into mainstream search, and Google has also expanded Search and Shopping ads within AI Overviews, clearly linking commercial discovery to AI-mediated answers.

Standalone LLM platforms face similar challenges. OpenAI has publicly stated that it began testing ads in ChatGPT in the United States on February 9, 2026, for users logged into both Free and Go plans, with ads clearly labelled and separated from the main answer [5]. OpenAI also claims that ads do not influence ChatGPT’s answers and that conversations with users are not provided to advertisers [5].

Unlike traditional search engines, LLM outputs are non-deterministic and shaped by patterns across vast bodies of information. This creates an opportunity for ‘LLM poisoning’, whereby large volumes of biased or self-promoting content are introduced into the information ecosystem to influence generated outputs. A sufficiently resourced individual or organisation could flood the internet with search-optimised articles, reviews, embellished achievements and AI-generated content, creating the appearance of widespread endorsement and authority. Through scale rather than accuracy, such content could disproportionately influence generated responses. Importantly, this occult manipulation may not be visible to either patients or clinicians, as outputs appear as coherent, neutral summaries rather than ranked sources.

Patients seeking ophthalmic evaluation and treatment commonly self-refer, seek second opinions, and make decisions influenced by accessibility, perceived expertise, price and reputation. A patient might ask an LLM, “who is the best retinal surgeon in Australia?” or “Where should I go for cataract surgery?”. Such questions are never purely objective. Although UK regulations place strict limits on comparative advertising, including prohibiting claims of superiority over identifiable alternatives in healthcare contexts, digital presence can be manipulated to make LLM outputs perform such comparisons indirectly [6].

These implications extend beyond clinical choice. A person with suspected optic neuritis may ask which MRI centre has the best scanner, shortest wait, or cheapest price. A patient with transient visual loss might ask whether neuroimaging is needed at all. These questions previously led users to compare multiple sources. In a conversational AI setting, a single convincing answer may funnel patients toward a particular clinician, imaging modality, or interpretation of urgency. In specialties such as ophthalmology where diagnostic timing materially affects outcomes, this shift is far from trivial [7, 8].

Treatment questions raise a further concern. If a patient asks, “What is the best treatment for multiple sclerosis?” or “Does steroid X have a role in optic neuritis?”, the generated answer may influence what they request, resist, or expect from the consultation. Even when clinicians correct misunderstandings, the upstream influence on the consultation is likely to affect treatment choice. The effects may be more assertive requests for branded therapies, increased confidence in experimental treatments or misguided suspicion for standard, evidence-based care. The concern is not simply misinformation, but the normalisation of commercially framed suggestions at the point when patients expect ‘neutral’ synthesis.

AI systems have demonstrated proficiency and real-world evidence in ophthalmic workflows. Recent studies have assessed chatbot performance in ophthalmic triage, diagnosis and referral pathways, and have shown that such systems can significantly shape ophthalmic practice [9–11]. As LLM interfaces become clinically integrated, commercial influence may subtly permeate into medical decision support layers already used by clinicians.

SEO influences are easy to overlook when conversational AI systems feel efficient and comprehensive [12]. As the fluency of LLM generated answers increases, the less visible the underlying contest for attention becomes. What patients may interpret as an expert consensus can be shaped by underlying data biases, system-level design choices and commercial influences [13].

Advertising in LLMs is not inherently incompatible with medicine but urgent questions arise: How should sponsored material be labelled in health-related prompts? How should conflicts of interest be declared in AI-mediated recommendations, and what regulatory frameworks are appropriate when conversational systems function as de facto decision-support tools? What safeguards are necessary when clinical relevance and commercial interests diverge?

These questions are not simply theoretical. They are already influencing how patients seek clinicians, what investigations they seek, and how they arrive at a consultation with pre-conceived beliefs and expectations [13, 14]. As advertising encroaches upon the step at which patients first understand information, the collision of commercial influence and clinical care moves closer to the moment of clinical decision-making.

Addressing these challenges requires coordinated action. Clinicians and patients should be aware that LLM outputs are influenced by commercial and informational biases. LLM platforms must improve transparency, including source attribution and fact-checking tools to enable human verification. Regulators should explore and implement mechanisms to remove misinformation and impose penalties on those responsible. This remains an almost insurmountable task, as if a single clinician manipulates peers, patients and the broader information ecosystem in ways that cannot be proven, accountability may be impossible to enforce.

The risk is no longer limited to passive commercial influence, but extends to active manipulation of the information substrate from which LLMs derive their answers. What appears to patients as neutral, expert synthesis may instead reflect engineered prominence rather than clinical merit. The question is no longer whether AI will influence medical decision-making, but whether that influence can be protected from direct manipulation.
